# PET/CT guidance for percutaneous fine needle aspiration cytology/biopsy

**DOI:** 10.4103/0971-3026.54885

**Published:** 2009-08

**Authors:** MJ Govindarajan, KR Nagaraj, KG Kallur, PS Sridhar

**Affiliations:** Consulting Radiologist, Bangalore Institute of Oncology – Health Care Global, Bangalore, India; 1 Consulting Nuclear Medicine Specialist, Bangalore Institute of Oncology – Health Care Global, Bangalore, India; 2 Consulting Radiation oncologist, Bangalore Institute of Oncology – Health Care Global, Bangalore, India

**Keywords:** PET/CT, tumor, FNAC/biopsy

## Abstract

PET/CT, used as a guiding tool, can improve the accuracy of percutaneous fine needle aspiration cytology (FNAC)/biopsy due to its ability to incorporate both physiological and anatomical information.

## Introduction

One of the uses of PET/CT fusion imaging, apart from cancer detection, staging and restaging and non-neoplastic applications in cardiac and neurological diseases, [1–4] is in providing guidance during percutaneous biopsy; this application of PET/CT has not been exhaustively studied. We would like to describe our limited experience in performing PET/CT-guided biopsies.

## Technical Note

Although there are no definite described methods in the literature, we believe, there can be, for all practical purposes, at least two ways of using PET/CT for percutaneous biopsy guidance.

**Figure 1 (A,B):**
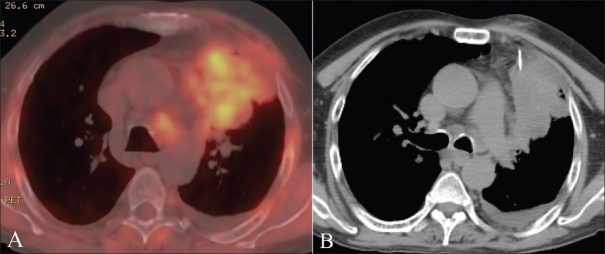
Axial fused PET/CT chest image in a 60-year-old man demonstrates a large left lung mass with relatively little metabolic activity in the easily accessible peripheral area. Plain CT scan (B) at the same level shows the lack of distinction between the metabolically active and inactive regions. The needle (arrow) is directed toward the metabolically more active focus. The diagnosis was carcinoma

**Figure 2 (A-C):**
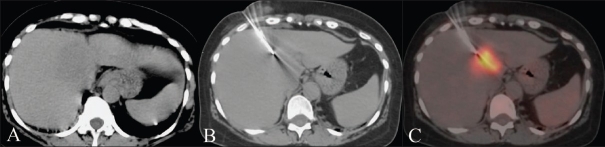
Noncontrast CT scan of the liver (A) during a percutaneous biopsy showing an area of subtle hypodensity which is nearly indistinct from the rest of the liver parenchyma. CT scan (B) shows the needle in place. The accuracy of positioning in the metabolically active lesion is confirmed on the fused PET/CT image (C). The diagnosis was an inflammatory lesion

**Figure 3 (A-C):**
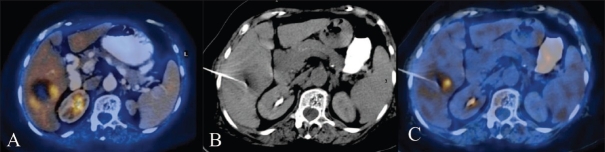
Axial fused PET/CT image in a 57-year-old woman demonstrates a necrotic mass in the right lobe of the liver and a small focus of increased metabolic activity along the wall, which is more along the medial aspect. CT scan (B) shows the needle tip in place. The accuracy of positioning of the needle tip in the most metabolically active medial portion is confirmed on the axial fused PET/CT image. The diagnosis was hepatoma

The first is where information from a PET scan done previously is used to target a metabolically active lesion or lesion-part[5] so that the diagnostic yield improves [Figure 1]. The second is where the biopsy is done immediately after a PET/CT study, without changing the patient's position [Figures 2 and 3]. We have now performed nine biopsies using the second method. In this method, the PET/CT is first performed in the usual manner. Using this information, the appropriate site to be biopsied is selected and the procedure is started with CT guidance. The CT scan image acquired during and after the needle insertion is fused with PET images acquired before needle insertion to confirm that the needle tip is in the right place. The advantages of this technique are:

- real-time confirmation that the needle tip is correctly positioned;
- the radiologist is more confident about biopsying the most metabolically active part of the lesion

The disadvantages of this technique are:

- it takes more time than traditional techniques, since fusion is necessary, which takes a few more seconds per image;
- we use a lead shield to reduce radiation dose to the operator, which limits the movements of the radiologist, and
- radiation exposure to the operator and supporting staff is more.

Anecdotally, we believe that the results of this technique are superior to traditional techniques and larger studies are required to confirm this.

## Conclusions

PET/CT-guided biopsies may help in difficult situations, especially when it is important to know which part of the tumor is active or which lesion is active in patients with multiple, widespread lesions.
